# Comparative effectiveness of incretin-based therapies and the risk of death and cardiovascular events in 38,233 metformin monotherapy users

**DOI:** 10.1097/MD.0000000000003995

**Published:** 2016-07-01

**Authors:** John-Michael Gamble, Jamie M. Thomas, Laurie K. Twells, William K. Midodzi, Sumit R. Majumdar

**Affiliations:** a School of Pharmacy; b Faculty of Medicine, Memorial University of Newfoundland, St. John's, Newfoundland and Labrador, A1B 3V6; c Division of General Internal Medicine, Department of Medicine, University of Alberta, Edmonton, Alberta, T6G 2B7, Canada.

**Keywords:** antidiabetic drugs, cardiovascular diseases, cohort studies, dipeptidyl-peptidase IV inhibitors, mortality, sulfonylurea compounds, type 2 diabetes mellitus

## Abstract

There is limited comparative effectiveness evidence to guide approaches to managing diabetes in individuals failing metformin monotherapy. Our aim was to compare the incidence of all-cause mortality and major adverse cardiovascular events (MACEs) among new metformin monotherapy users initiating a dipeptidyl-peptidase-4 inhibitor (DPP4i), glucagon-like peptide-1 receptor agonist (GLP-1RA), sulfonylurea (SU), thiazolidinedione, or insulin.

We conducted a cohort study using the UK-based Clinical Practice Research Datalink. Participants included a cohort of 38,233 new users of metformin monotherapy who initiated a 2nd antidiabetic agent between January 1, 2007 and December 31, 2012 with follow-up until death, disenrollment, therapy discontinuation, or study end-date. A subcohort of 21,848 patients with linked hospital episode statistics (HES) and Office of National Statistics (ONS) data were studied to include MACE and cardiovascular-related death. The primary exposure contrasts, defined a priori, were initiation of a DPP4i versus an SU and initiation of a GLP-1RA versus an SU following metformin monotherapy. Cox proportional hazards models were used to assess the relative differences in time to mortality and MACE between exposure contrasts, adjusting for important baseline patient factors and comedications used during follow-up.

The main study cohort consisted of 6213 (16%) patients who initiated a DPP4i, 25,916 initiated an SU (68%), 4437 (12%) initiated a TZD, 487 (1%) initiated a GLP-1RA, 804 (2%) initiated insulin, and 376 (1%) initiated a miscellaneous agent as their 2nd antidiabetic agent. Mean age was 62 years, 59% were male, and mean glycated hemoglobin was 8.8% (92.6 mmol/mol). Median follow-up was 2.7 years (interquartile range 1.3–4.2). Mortality rates were 8.2 deaths/1000 person-years for DPP4i and 19.1 deaths/1000 person-years for SU initiators. Adjusted hazards ratio (aHR) for mortality in DPP4i versus SU initiators = 0.58, 95% CI 0.46 to 0.73, *P* < 0.001. MACE rates were 19.1/1000 person-years for DPP4i initiators, 15.9/1000 person-years for GLP1-RA initiators versus 33.1/1000 person-years for SU initiators (aHR: DPP4i vs SU initiators = 0.64, 95%CI 0.52–0.80; GLP1RA vs SU initiators = 0.73, 95% CI 0.34–1.55).

In this cohort of metformin monotherapy users, 2nd-line DPP4i use was associated with a 42% relative reduction in all-cause mortality and 36% reduction in MACE versus SUs, the most common 2nd-line therapy in our study. GLP-1RAs were not associated with adverse events in this cohort.

## Abstract 2

Supplemental Digital Content is available in the text

## 1 Introduction

Current clinical practice guidelines recommend initiating metformin 1st in all patients with type 2 diabetes without a contraindication such as reduced estimated glomerular filtration rate (eGFR) or a history of lactic acidosis.^[1]^ However, because type 2 diabetes is characterized by a progressive loss of pancreatic β-cell function, most individuals eventually require multiple antidiabetic medications. Within 2-years of initiating metformin monotherapy, over 30% of patients require an additional antidiabetic agent and within 3-years over 50% require combination therapy.^[2]^ Given the lack of evidence for long-term effectiveness in preventing diabetes-related morbidity and mortality and concerns over the safety of newer antidiabetic agents, it is unclear how to optimally manage patients who fail on metformin therapy.^[3]^ To date, there is a paucity of comparative effectiveness evidence on the long-term clinical outcomes for most antidiabetic agents in general, and the incretin-based therapies in particular.

The incretin-based therapies, namely dipeptidyl-peptidase-4 inhibitors (DPP4is) and glucagon-like peptide-1 receptor agonists (GLP-1RAs), have potential advantages over other antidiabetic agents in terms of not provoking hypoglycemia or weight gain and perhaps being associated with pleiotropic benefits on the cardiovascular system.^[4]^ Preclinical studies indicate that GLP-1RAs are associated with improved cardiac function and reduced infarct size.^[5]^ Clinically, both DPP4i and GLP-1RA therapies modestly improve some cardiovascular risk factors including low-density lipoprotein, high-density lipoprotein, triglycerides, and blood pressure, although these improvements have not translated into demonstrable reductions in cardiovascular events. Furthermore, recent large placebo controlled trials have found no differences in mortality and major adverse cardiovascular events (MACEs) among alogliptin, saxagliptin, and sitagliptin users.^[6–9]^ Despite approximately 36,000 patients enrolled in these trials, strict study protocols not representative of typical care, exclusion of complex patients, limited treatment options, and placebo comparisons limit the generalizability of these studies and their usefulness for evaluating hypotheses about comparative safety and effectiveness of various contemporary treatments.

Thus, high-quality observational studies of comparative effectiveness are needed to complement the trials data and extend the evidence-base. To date, observational studies of the incretins evaluating mortality and cardiovascular outcomes have shown conflicting results, and are heterogeneous in design with variation in study cohorts, exposure definitions, and outcome definitions.^[10–16]^ Therefore, we sought to assess the overall and cardiovascular safety and effectiveness of incretin-based therapies compared to the current standard of care through a rigorous population-based cohort study using clinically rich and well-validated databases with extended follow-up periods.

## 2 Methods

### 2.1 Study design and data sources

This population-based cohort study used data from the UK-based Clinical Practice Research Datalink (CPRD) database, which captures electronic medical information for primary care encounters by general practitioners. Our source population consisted of all patients in the CPRD database that received a new prescription for any antidiabetic agent between January 1, 2001 and December 31, 2012. A subcohort of 21,848 patients (57%) selected from the full cohort was linked to hospital episode statistics (HES) and Office of National Statistics (ONS) data to capture events that occurred in hospital as well as cause of death information. Briefly, the CPRD database contains individual deidentified longitudinal data collected from over 650 primary care practices in the UK. The population covers approximately 7% of the UK population and is representative of the UK population.^[17]^ The available information includes patient sociodemographic data (e.g., deprivation index), health behaviors (e.g., smoking), physiological measures (e.g., blood pressure), laboratory data (e.g., glycated hemoglobin [A1c]), clinician-assigned diagnoses, and outpatient prescription records. Diagnoses are coded using the Read classification system within the CPRD database and using International Classification of Diseases Version 10 in the HES and ONS databases. Data validation is performed on an ongoing basis in accordance with standardized guidelines that certify practices as up-to-standard and over 350 validation studies have been performed.^[18,19]^ Details regarding the data quality, linkages, and utility are available elsewhere.^[20]^ Our study protocol was approved by the Independent Scientific Advisory Committee (ISAC 13_100R, August 2013) and received approval from the Health Research Ethics Board at Memorial University.

### 2.2 Study cohort

The study cohort was restricted to new users of metformin monotherapy who subsequently initiated a 2nd antidiabetic agent on or after January 1, 2007 as the 1st incretin-based therapies were approved by the European Medicines Agency in November 2006. Patients included in the main analysis 30 years of age and older with at least 12 months of up-to-standard medical history. “New users” were defined as patients with no prescription record for any antidiabetic drug, including insulin, for 365 days prior to the initial antidiabetic agent prescription. Women who were pregnant, had a history of gestational diabetes, or a diagnosis of polycystic ovary syndrome were excluded. Patients who died or left a CPRD practice prior to initiation of a 2nd antidiabetic agent were also excluded.

### 2.3 Exposure definitions

On the day a patient initiated a 2nd oral antidiabetic drug following metformin monotherapy, they began to contribute time at risk to 1 of 6 antidiabetic exposure groups of interest: sulfonylurea therapy (SU) [reference group], DPP4i therapy, GLP-1RA therapy, insulin therapy, thiazolidinedione (TZD) therapy, and miscellaneous antidiabetic therapy including acarbose and meglitinides (other [OTH]). Only a single prescription was required for patients to accrue time-at-risk in the aforementioned exposure categories, as there is no biological rationale why early events could not occur with short exposure duration. Patients contributed person time-at-risk regardless of treatment overlap with metformin therapy. A patient was considered as actively exposed for the duration of time they received a prescription for each antidiabetic drug, which was based on the quantity of drug prescribed and dosing instructions. To account for potential nonadherence, we included a portion of follow-up time following the end of the expected medication supply that was equivalent to 50% of the prescription duration. We assumed a minimum of 30-days exposure, and if the expected days’ supply was missing or implausible, then we assumed a 90-day exposure period.

### 2.4 Outcome definitions

Our co-primary endpoints, defined a priori, were all-cause mortality and MACE (composite of nonfatal myocardial infarction, non-fatal stroke, or any cardiovascular-related mortality). As cause specific mortality data (i.e., cardiovascular-related mortality) are only available in the subpopulation linked with HES/ONS, all analyses for the outcome of MACE or cause-specific mortality were restricted to this subpopulation.

Secondary endpoints included the individual components of our primary outcomes as well as other clinically important cardiovascular events: heart failure, unstable angina, urgent revascularizations, and cardiac arrhythmias including atrial fibrillation, atrioventricular block, ventricular and supraventricular tachycardias, cardiac arrest, and other unspecified conduction disorders. For all composite outcomes, we included only the 1st event after their index date as the dependent variable (failure time) in our analyses. Outcome definitions were based on previously validated Read codes contained in the CPRD data and International Classification of Diseases Version 10 codes within HES/ONS linked data.^[18,21]^

### 2.5 Statistical analysis

Baseline patient characteristics were described across the 6 antidiabetic exposure groups using appropriate summary statistics at the time of initiation of a patient's 2nd antidiabetic agent, defined as the index date. Patients contributed time at risk to one of the exposure groups of interest from their index date until: the 1st occurrence of an outcome of interest or the date a patient ceased to meet the definition of exposure or death or the date of emigration from a CPRD practice or the study end-date (October 1, 2013). The independent associations between the exposure groups of interest and outcomes were assessed using Cox proportional hazards regression models. Our primary exposure contrasts, defined a priori, were initiation of a DPP4i versus an SU and initiation of a GLP-1RA versus an SU following metformin monotherapy. Of note, for any outcomes where the cell sizes were <5, we did not conduct any further analyses. For example, given the small number of deaths (<5) that occurred in the initiators of 2nd-line GLP-1RA for our primary analysis, we did not report treatment effect estimates.

To adjust for potential confounding, numerous covariates were included in the multivariable Cox proportional hazards models. All covariates included in the model are listed in the footnotes of the tables and were defined a priori based on biological rationale, clinical experience, and availability within the CPRD data. Kidney function was estimated using an abbreviated modification of diet in renal disease equation.^[22]^ Values for smoking status, body mass index (BMI), A1c, systolic blood pressure, and eGFR were based on the most recent value prior to the index date, or if missing, the most recent value after the index date was used. Multiple imputation was used to handle missing data for smoking status (16.3% missing), BMI (1.3% missing), A1c (4.4% missing), and blood pressure (4.9% missing).

Furthermore, to account for patterns in antidiabetic use following initiation of 2nd-line therapy, we used time-dependent covariates to account for exposure to 3rd, 4th, 5th, 6th, and 7th line initiation of antidiabetic therapy by including dummy variables to classify exposure order to SU, DPP4i, GLP-1RA, insulin, TZD, and miscellaneous agents. In addition, we evaluated several prespecified potential effect modifiers including sex, age, history of cardiovascular disease, history of heart failure, presence of moderate to severe renal impairment (eGFR < 60 mL/min/1.73 m^2^), duration of treatment prior to index, BMI, and A1c.

### 2.6 Sensitivity analyses

We conducted several sensitivity analyses to assess the robustness of the results. First, we varied the reference group from SU to other commonly used antidiabetic agents including insulin and TZDs separately. Second, we repeated the analysis for all outcomes using the HES/ONS linked population only. Third, we varied our definition of exposure to continuous use whereby we censored follow-up time at the 1st prescription discontinuation for the index agent. Fourth, we censored follow-up time at the start of the 3rd antidiabetic agent, therefore restricting follow-up time to include exposure to metformin and 1 of the 6 antidiabetic categories of interest with no further antidiabetic add-on therapy. Fifth, we reframed our index date to the 1st date a patient initiated their 3rd antidiabetic agent either as a switch from or addition to their existing therapy. Sixth, we varied our analysis to categorize antidiabetic exposure throughout the entire follow-up period using time-dependent variables to categorize antidiabetic exposure, irrespective of which order initiation occurred following metformin monotherapy. Last, given the relatively high numbers of model covariates compared to outcomes, we used a propensity score analysis as an alternative method to adjust for potential confounding. Propensity scores were derived from a multivariable logistic regression model using all covariates, except age and sex, measured prior to the index date. Deciles of the propensity score, age, and sex were included as covariates in the multivariable Cox proportional hazards models used to quantify adjusted hazard ratios (aHRs) for the exposure-outcome associations of interest. All analyses were conducted with Stata 13/MP (StataCorp LP, Stata Statistical Software: Release 12. College Station, TX).

## 3 Results

**Figure 1:**
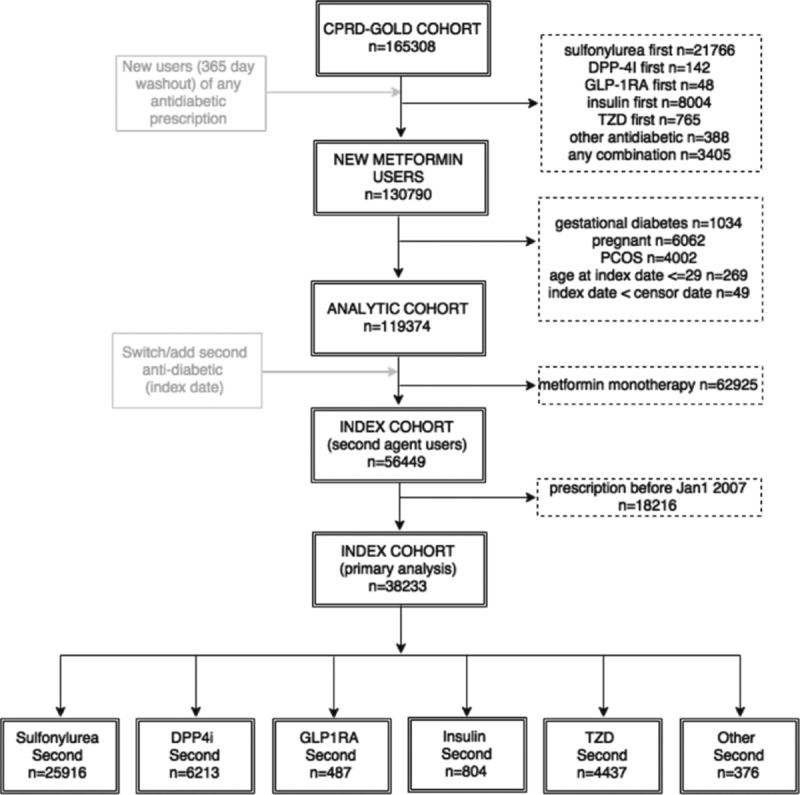
Schematic depicting the flow of Clinical Practice Research Datalink (CPRD) patients in the cohort.

The main study cohort comprised 38,233 metformin monotherapy users who started a 2nd antidiabetic agent after January 1, 2007, (Figure 1) of which 6213 (16%) initiated a DPP4i; 487 (1%) a GLP-1RA; 25,916 an SU (68%); 4437 (12%) a TZD; 804 (2%) an insulin; and 376 (1%) an OTH. Baseline population characteristics are shown in Table 1. The median duration of metformin monotherapy prior to initiation of the 2nd antidiabetic agent was 2.3 years (interquartile range [IQR] 0.55–3.5) and the median person-years follow-up after the initiation of the 2nd antidiabetic prescription date was 2.7 years (IQR 1.3–4.2). Mean age was 62 years, 59% were male, 13.8% had prior cardiovascular disease, 15.4% had an eGFR less than 60 mL/min/1.73 m^2^, mean BMI was 32.4 kg/m^2^, and mean A1c was 8.8%.

**Table 1:**
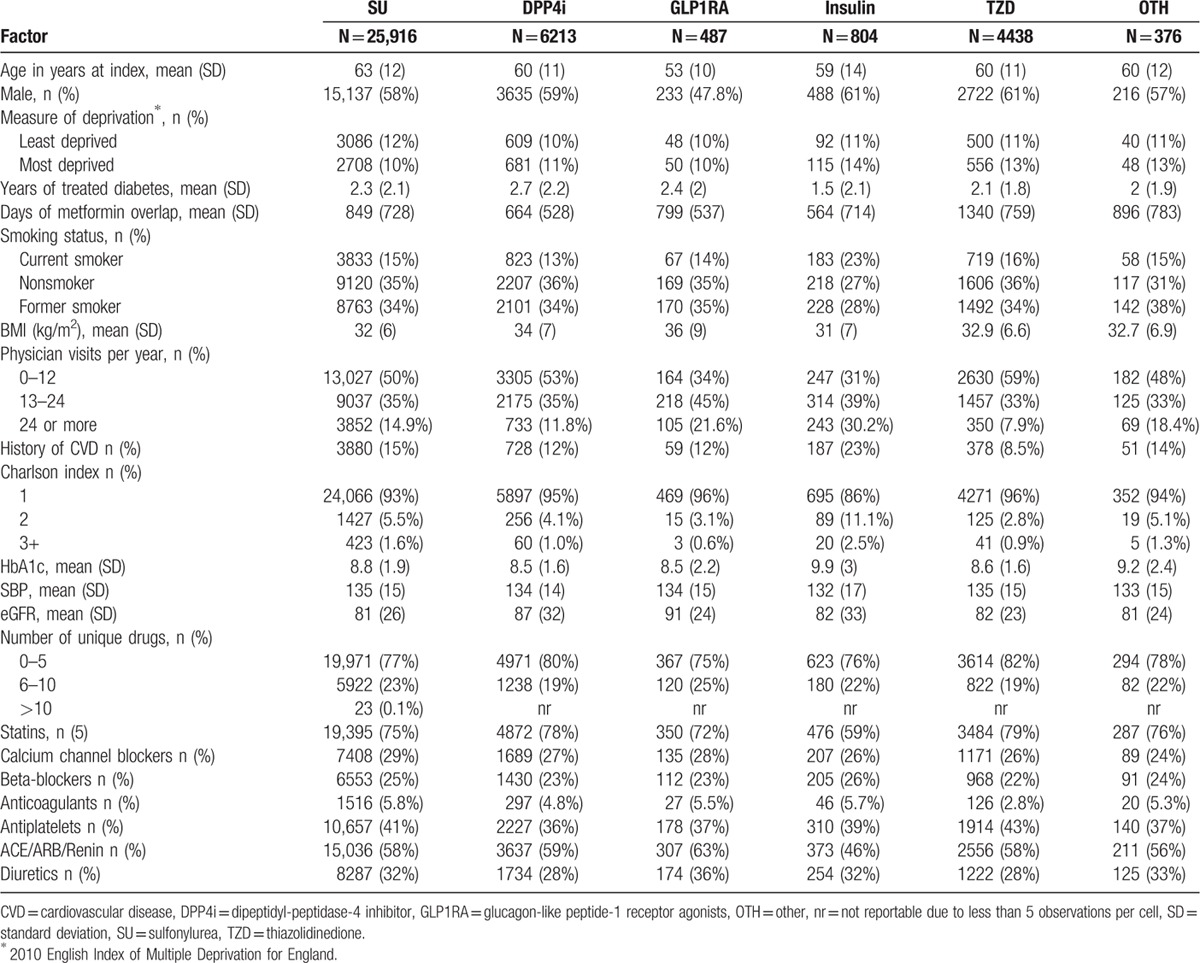
Baseline characteristics of new-users of 2nd-line antidiabetic therapies in a cohort of new metformin users (N = 38,233).

DPP4i initiators were on average younger (60 vs 63 years), used metformin monotherapy for longer (2.7 vs 2.3 years), had a higher BMI (34 vs 32 kg/m^2^), and had a lower A1c (8.5% vs 8.8%), compared to SU initiators; similar differences were seen with GLP-1RA initiators (Table 1). A total of 21,848 (57%) patients were linked to hospitalization (HES) and death certificate (ONS) data of which 15% initiated DPP4i, 1% GLP-1RA, 69% SU, 12% TZD, 2% insulin, and 1% OTH (Supplemental Figure 1 and Supplemental Table 1).

### 3.1 All-cause mortality

There were a total of 2684 deaths in the study period, 1500 of those events occurred while patients were exposed to their 2nd antidiabetic agent following metformin monotherapy. Mortality rates were 8.2 deaths per 1000 person-years (95% confidence interval [CI] 6.6–10.1) for DPP4i initiators, 19.1 deaths per 1000 person-years (95% CI 18.0–20.2) for SU initiators, 7.2 deaths per 1000 person-years (95% CI 5.8–8.9) for TZD initiators, 70.9 deaths per 1000 person-years (95% CI 59.1–85.0) for insulin initiators, and 18.7 deaths per 1000 person-years (95% CI 11.4–30.5) for OTH (Table 2). After adjusting for potential confounders, among patients who started metformin monotherapy, the initiation of a DPP4i compared to an SU was associated with a significant 42% reduction in mortality (absolute rate difference = 11 events per 1000 person-years, aHR = 0.58, 95% CI 0.46–0.73, *P* < 0.001) (Table 2).

**Table 2:**
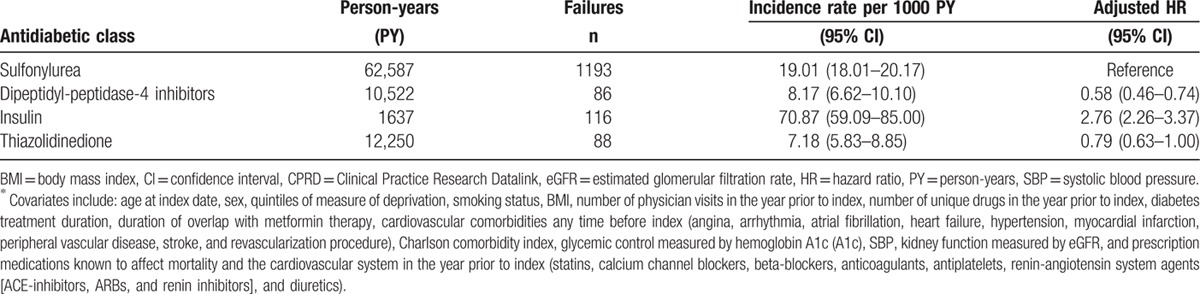
All-cause mortality rates and adjusted^∗^ HRs for patients from the CPRD base population (N = 38,233) initiating 1 of the 6 antidiabetic classes following metformin monotherapy.

### 3.2 Major adverse cardiovascular events

Over the study period, there were a total of 1854 MACE that occurred within the HES and ONS linked population (n = 21,848), 1455 of those events occurred in patients that initiated a 2nd-line antidiabetic agent. For DPP4i initiators, there were 19.1 MACE per 1000 person-years (95% CI 15.7–23.3); for GLP-1RA initiators, 15.9 MACE per 1000 person-years (95% CI 7.6–33.4); for SU initiators, 33.1 MACE per 1000 person-years (95% CI 31.2–35.1); for TZD initiators, 20.7 MACE per 1000 person-years (95% CI 17.5–24.5); for insulin initiators, 63.3 MACE per 1000 person-years (95% CI 48.7–82.3); and for initiators of OTH, 28.4 MACE per 1000 person-years (95% CI 16.8–47.9) (Table 3). Compared to 2nd-line SU use, 2nd-line DPP4i use was associated with a statistically significant reduction in MACE (absolute risk difference = 14 events per 1000 person-years, aHR = 0.64, 95% CI 0.52–0.80, *P* < 0.001). GLP1RAs were not associated with a reduction in MACE (absolute risk difference = 17 events per 1000 person-years, aHR = 0.73, 95% CI 0.34–1.55, *P* = 0.44).

**Table 3:**
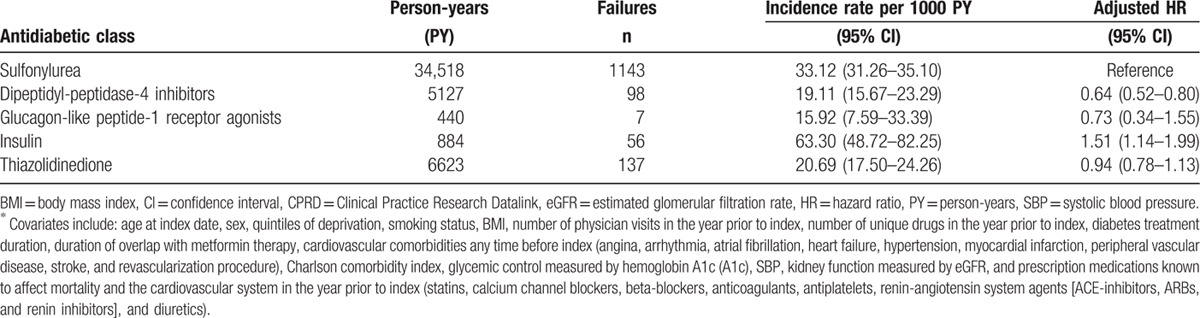
MACE incidence and adjusted^∗^ HRs for patients from the HES/ONS linked population (N = 21,848) initiating one of the six antidiabetic classes following metformin monotherapy.

### 3.3 Secondary outcomes

**Figure 2:**
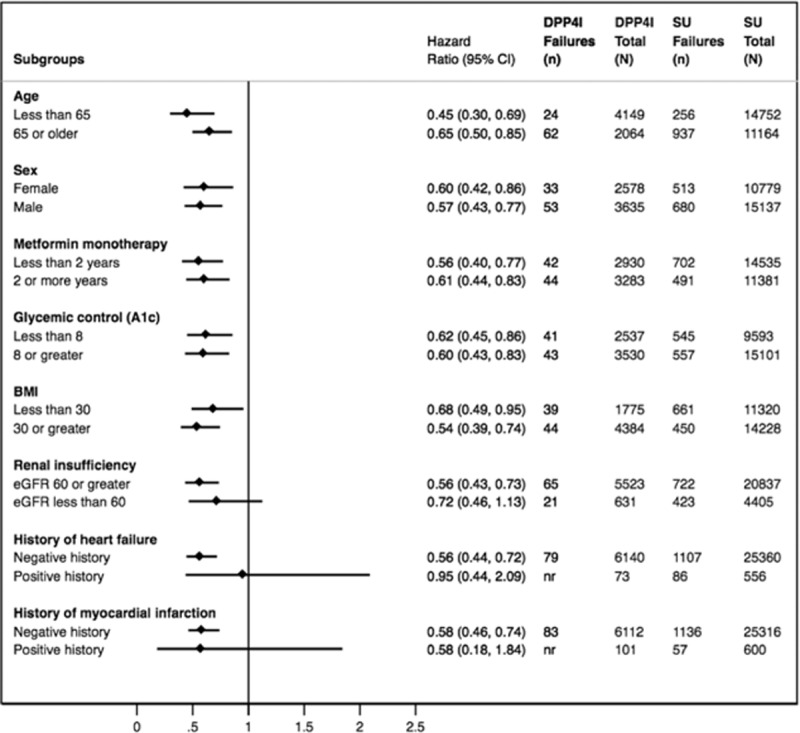
Adjusted Cox model subgroup analysis of all-cause mortality in patients using 2nd-line DPP4i after metformin monotherapy compared to 2nd-line SU. DPP4i = dipeptidyl-peptidase-4 inhibitor, SU = sulfonylurea.

Initiators of a DPP4i as a 2nd-line agent compared to SU were associated with a statistically significant reduction in risk for 5 of the 6 secondary outcomes: unstable angina (aHR = 0.64 95% CI 0.52–0.80), arrhythmia (aHR = 0.66 95% CI 0.55–0.78), heart failure (aHR = 0.57 95% CI 0.42–0.75), myocardial infarction (aHR = 0.66 95% CI 0.47–0.94), and urgent revascularization (aHR = 0.58 95% CI 0.52–0.64), but not stroke (aHR = 0.97 95% CI 0.80–1.17). There was also a significant reduction in risk for cardiovascular death (absolute rate difference = 6 events per 1000 person-years, aHR = 0.16, 95% CI 0.06–0.44). There were no significant subgroup treatment effects observed for a priori defined patient characteristics hypothesized to have a potential impact on the risk of mortality (*P*-value > 0.15 for all interaction terms) (Figure 2 and Supplemental Figure 2).

### 3.4 Sensitivity analysis

**Figure 3:**
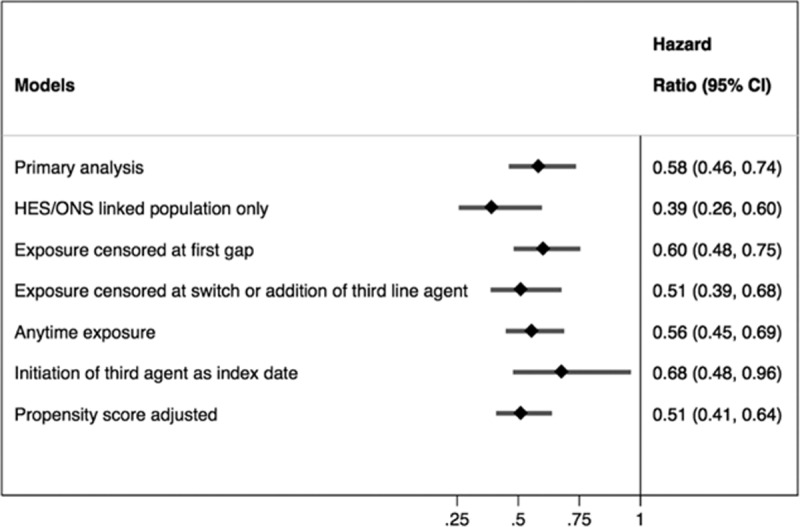
Adjusted hazard ratio and 95% confidence interval from sensitivity anlayses for mortality in DPP4i users versus SU users. DPP4i = dipeptidyl-peptidase-4 inhibitor, nr = not reportable, SU = sulfonylurea.

All sensitivity analyses supported the robustness of our primary findings (Figure 3**)**. A significant reduction in mortality was also found when 2nd-line DPP4i users were compared to 2nd-line insulin users (aHR = 0.21, 95% CI 0.16–0.28, *P* < 0.001) and with 2nd-line TZD (aHR = 0.74, 95% CI 0.54–0.99, *P* = 0.04). Anytime use of a DPP4i after metformin monotherapy was also associated with a reduction in mortality compared to SU use (aHR = 0.56, 95% CI 0.45–0.69). Anytime use of a GLP1RA was not associated with a difference in mortality (aHR = 0.70, 95% CI 0.38–1.27). Statistically significant differences in MACE were observed for 2nd-line DPP4i compared to 2nd-line insulin (aHR = 0.43, 95% CI 0.30–0.60, *P* < 0.001) and 2nd-line TZD (aHR = 0.68, 95% CI 0.52–0.89, *P* = 0.005) users. Accounting for anytime use of a DPP4i after metformin monotherapy compared to anytime use of an SU, a significant reduction in the risk of MACE was observed (aHR = 0.67, 95% CI 0.55–0.82, *P* < 0.001). Anytime exposure of a GLP1RA was not associated with a reduction in MACE (aHR = 0.75, 95% CI 0.46–1.24).

## 4 Discussion

### 4.1 Main results

Using a large population-based cohort of new-users of metformin monotherapy, we found that initiation of 2nd-line DPP4i was associated with a 42% reduction in mortality and 36% reduction in the composite of myocardial infarction, stroke, or cardiovascular death compared to 2nd-line initiation of SU. Similarly, 2nd-line DPP4i use was associated with a reduction in death and cardiovascular events compared to 2nd-line insulin and TZD use. With the exception of stroke (no association), DPP4is were also associated with a decreased risk of each individual cardiovascular endpoint examined. Second-line initiation of GLP-1RAs was not associated with an increased or decreased risk of MACE compared to SU therapy.

These findings are consistent with the putative cardioprotection reported in preclinical and clinical studies of incretins.^[5,23,24]^ Alternatively, our findings could be a result of adverse cardiovascular effects of the SU comparator antidiabetic therapies we studied. The cardiotoxicity of SUs has been an area of considerable debate for over 40 years and is an area of continued investigation.^[25]^ Mechanisms by which SUs may affect cardiovascular risk include attenuation of ischemic preconditioning, hyperinsulinemia, weight gain, and hypoglycemia. However, in sensitivity analyses where the comparator groups were insulin or TZDs, the findings were similar suggesting to us that cardioprotection may be the more likely explanation.

### 4.2 Comparison with other literature

Recent randomized controlled trials have found no statistical or clinical differences between DPP4is and placebo for the incidence of major cardiovascular outcomes including myocardial infarction, stroke, or cardiovascular death.^[6–8]^ Likewise, a recent cardiovascular outcome trial, ELIXA, found no differences in the rate of major adverse cardiovascular events for the GLP-1RA lixisenatide compared to placebo.^[26]^

Previous observational studies evaluating DPP4is have been inconsistent in their findings.^[10–16,27]^ For example, using a large US-based claims and integrated laboratory database, Eurich et al^[10]^ conducted a cohort study including 72,738 new-users of oral antidiabetic drugs of which 8032 used sitagliptin. They found no difference in the risk of their primary composite outcome of all-cause mortality or hospitalization for those exposed to sitagliptin versus other antidiabetic drugs (aHR = 0.98, 95% CI 0.91–1.06). There were 32 deaths in the sitagliptin group thereby limiting the power to detect relative differences in the rate of death compared to nonsitagliptin users. A time-varying approach was used to categorize antidiabetic drug exposure over the entire observation period that assumes exchangeability of exposure categories irrespective of ordering or gaps in antidiabetic exposure. Conversely, Rathman et al^[11]^ used the German Disease Analyzer database (IMS HEALTH) and found new use of DPP4i was associated with a decreased risk of macrovascular events (aHR = 0.74, 95% CI 0.67–0.82), defined as primary care diagnoses for coronary heart disease, myocardial infarction, stroke, and peripheral vascular disease; and Ou et al^[16]^ used administrative data from Taiwan and found DPP4is were associated with decreased mortality (aHR = 0.63, 95% CI 0.55–0.72), MACE (aHR = 0.63, 95% CI 0.55–0.83), and ischemic stroke (aHR = 0.43, 95% CI 0.51–0.81) but no difference in the risk of myocardial infarction.

In fact, there have been 3 prior studies using the CPRD database to examine this important clinical question (using different analytic methods and smaller cohorts than ours), reporting inconsistent effects of DPP4i.^[12–14]^ The 1st study included 27,457 new metformin monotherapy users with type 2 diabetes who initiated a 2nd antidiabetic therapy. At least 180 days of metformin exposure was an inclusion criterion, creating a period of immortal time. They found reported combination MET and DPP4i (n = 1455) therapy was not associated with all-cause mortality (aHR = 0.61, 95% CI 0.29–1.30) or a composite of a myocardial infarction or stroke (aHR = 1.02, 95% CI 0.50–2.12).^[12]^ There were, however, only 9 deaths, 4 myocardial infarctions, and 4 strokes, in the metformin and DPP4i group versus 86 deaths and 98 MACE events in our study. The 2nd CPRD study used different inclusion criteria and exposure definitions and found a statistically significant decrease in mortality (aHR = 0.74, 95% CI 0.58–0.92) and nonsignificant decrease in MACE (aHR = 0.76, 95% CI 0.47–1.20) for MET-DPP4i users compared to MET-SU users. The 3rd study, by Yu et al,^[14]^ found the use of a DPP4i with metformin was associated with a reduction in the risk of a composite of all-cause mortality, myocardial infarction, or stroke (aHR = 0.62, 95% CI 0.40–0.98) compared to combination use of metformin an SU. However, this study was also limited in the number of events (12 MACE and 13 deaths in the Metformin-DPP4i group) and did not include cardiovascular-related death within their MACE composite.

Similarly, observational studies have shown mixed results regarding the association between GLP-1RA use and cardiovascular events.^[28–31]^ Although we had limited statistical power to evaluate the association between GLP-1RAs and cardiovascular events, our results are consistent with findings from other studies which found GLP-1RAs were not associated with an increased or decreased risk of cardiovascular events compared to SUs. Patorno et al^[28]^ used a large US commercial database to compare the incidence of a composite cardiovascular endpoint (hospitalization for acute MI, unstable angina, stroke, and coronary revascularization) between GLP-1RA initiators and SU initiators and found no differences in risk (aHR = 0.86, 95% CI 0.69–1.08). Using a Danish database, Mogensen et al^[29]^ also found no differences in cardiovascular risk between GLP-1RAs and SUs (aHR = 0.82, 95% CI 0.55–1.21). However, others have reported cardioprotective effects of GLP-1RAs, most likely due to differences in comparator groups and other methodological differences. For example, Paul et al^[31]^ used insulin as a comparator group, and found the GLP1RA, exenatide, was associated with a decreased risk of heart failure, MI, and stroke. Best et al^[30]^ also found exenatide was associated with a decreased risk of cardiovascular disease, however they used a comparator group with mixture of antidiabetic agents. All of the aforementioned studies lacked sufficient power to evaluate individual cardiovascular endpoints with precision.

How does our study add to this evidence? First, our design precludes immortal time bias.^[32]^ Second, we had many more events and a larger population with longer follow-up time. Third, our study includes a large number of outcomes for all-cause mortality and major adverse cardiovascular outcome events in DPP4i users, thereby providing sufficient power to minimize the risk of a type 2 statistical error. Importantly, our definition of MACE includes cardiovascular related mortality using death certificate data through linkage with ONS data. Finally, our study used the most clinically relevant study population – guideline concordant new metformin users who then initiated 1 of 6 widely available 2nd-line antidiabetic agents.

## 5 Limitations

Although our cohort study used rigorous design and analytic methods, several limitations must be acknowledged. First, as in all observational studies we cannot rule out the potential for residual confounding, or unknown or unmeasured confounders (e.g., ethnicity, family history, and activity level) as alternate explanation for our findings; however, we adjusted for numerous potential confounders (i.e., A1c, smoking, deprivation index, BMI, SBP, eGFR, etc.) that are often not available within computerized administrative databases. Second, confounding by indication is always a concern when evaluating intended outcomes of drug therapies using observational study designs. We used several design and analysis techniques to minimize this bias including restriction of subject to metformin monotherapy users, a new user design, an active comparator, and rigorous methods to control for confounding. Similarly, channeling may occur whereby patients differ in certain characteristics (e.g., comorbidities) that may affect decision to prescribe on drug versus another. Certainly, an individual's weight, financial status, comorbidities, and kidney function among other characteristics would be expected to influence whether an incretin-based therapy, insulin, TZD, or an SU was prescribed. Third, we assume that drug prescription is a surrogate marker for consumption. We recognize this method may overestimate actual exposure since primary and secondary adherence failure cannot be assessed and this may bias our observation towards the null hypothesis.

## 6 Conclusions

Our findings suggest that the guideline-concordant initiation of 2nd-line DPP4i following metformin monotherapy is associated with lower risk of all-cause mortality and major cardiovascular events compared to 2nd-line SU. It is noteworthy that almost 70% of the population received an SU as add-on therapy in our cohort. Until adequately powered active comparator trials of 2nd-line antidiabetic agents in patients on metformin monotherapy are reported, the evidence to date and now including our study suggests that DPP4is are the safest of the 2nd-line agents to use following failure of metformin monotherapy. Whether our findings are a result of a protective effect of DPP4i or a harmful effect of SUs cannot be determined by this study design, but the relative mortality reductions seen compared to other antidiabetic agents tends to favor the former.

## Supplementary Material

Supplemental Digital Content
