# Supplementary material for: Comparative effectiveness of incretin-based therapies and the risk of death and cardiovascular events in 38,233 metformin monotherapy users
Source: Medicine (Baltimore). 2016 Jul 1;95(26):e3995. doi: 10.1097/MD.0000000000003995 (PMC4937919; doi:10.1097/MD.0000000000003995)
Supplement: Supplemental Digital Content [file medi-95-e3995-s001.doc]

**SUPPLEMENTAL FIGURE 1.** Schematic depicting the flow of CPRD patients in the cohort.


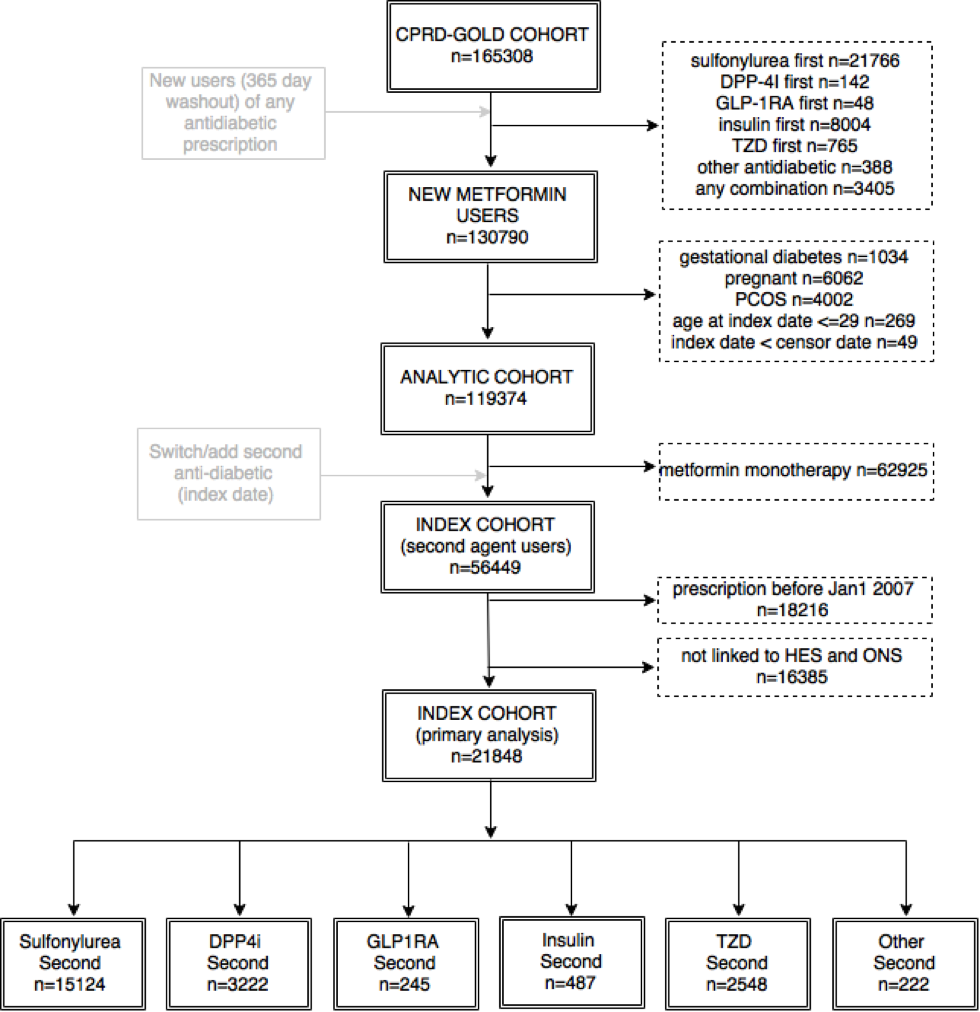


**SUPPLEMENTAL FIGURE 2.** Adjusted Cox model subgroup analysis of MACE in patients using second-line DPP4i after metformin monotherapy compared to second-line SU.


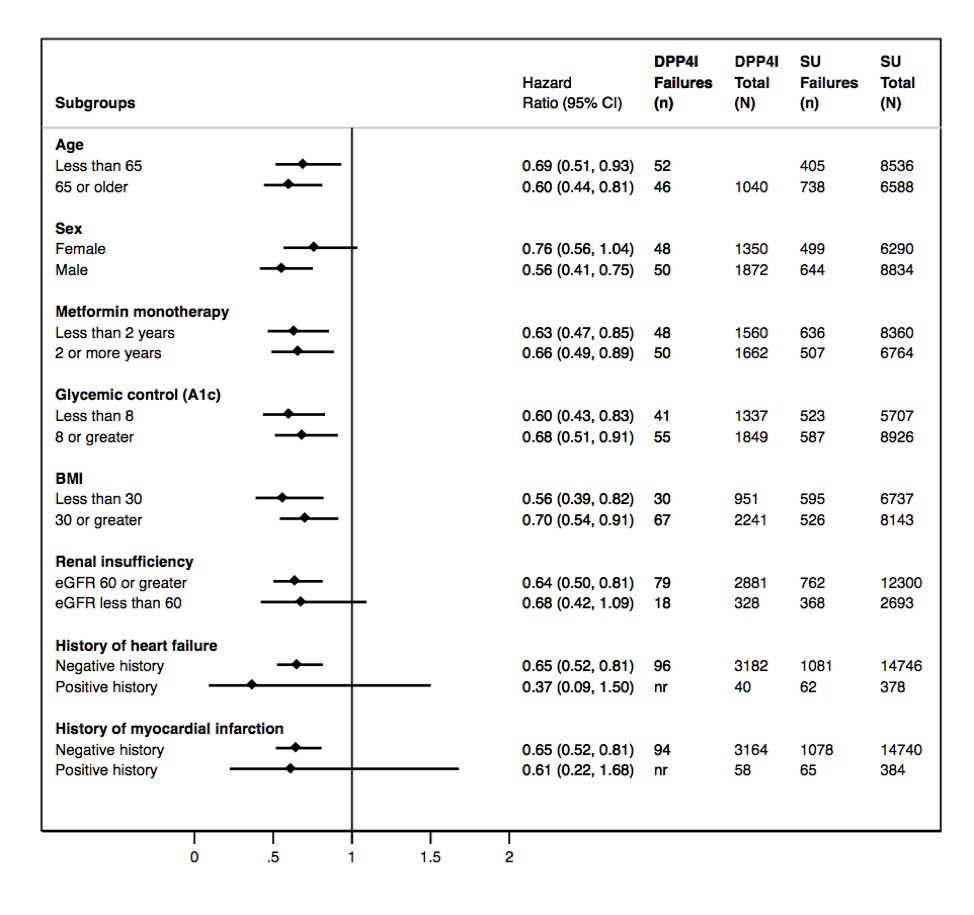


**SUPPLEMENTAL TABLE 1.** Baseline characteristics of patients with data linked to HES and ONS in the index cohort used in the primary analysis (N = 21848).

| **Factor**  N (%), unless specified | **SU** | **DPP4i** | **GLP1RA** | **INSULIN** | **TZD** | **OTH** |
| --- | --- | --- | --- | --- | --- | --- |
|  | N=15125 | N=3222 | N=245 | N=487 | N=2548 | N=222 |
| Age at Index, mean (SD) | 63.2 (12.4) | 60.1 (11.4) | 53.2 (10.1) | 60.0 (13.9) | 59.6 (11.3) | 59.9 (12.2) |
| Male | 8835 (58.4%) | 1872 (58.1%) | 123 (50.2%) | 312 (64.1%) | 1565 (61.4%) | 131 (59.0%) |
| Duration of Treated Diabetes in Days, mean (SD) | 2.3 (2.2) | 2.7 (2.3) | 2.5 (2.0) | 1.5 (2.1) | 2.2 (1.9) | 1.8 (1.9) |
| Metformin Overlap in Days, mean (SD) | 846.4 (721.6) | 647.4 (513.2) | 799.9 (512.5) | 544.6 (712.6) | 1310.5 (754.8) | 913.6 (799.8) |
| Smoking Status |  |  |  |  |  |  |
| Unknown | 2504 (16.6%) | 585 (18.2%) | 45 (18.4%) | 97 (19.9%) | 392 (15.4%) | 39 (17.6%) |
| Current | 2123 (14.0%) | 411 (12.8%) | 22 (9.0%) | 113 (23.2%) | 402 (15.8%) | 35 (15.8%) |
| Non | 5292 (35.0%) | 1133 (35.2%) | 85 (34.7%) | 128 (26.3%) | 905 (35.5%) | 68 (30.6%) |
| Former | 5206 (34.4%) | 1093 (33.9%) | 93 (38.0%) | 149 (30.6%) | 850 (33.3%) | 80 (36.0%) |
| Body Mass Index (kg/m^2^), mean (SD) | 31.6 (6.3) | 33.8 (6.5) | 42.7 (7.9) | 30.3 (7.7) | 32.7 (6.2) | 33.3 (7.7) |
| Physician Visits |  |  |  |  |  |  |
| 0 to 12 | 7846 (51.9%) | 1763 (54.7%) | 91 (37.1%) | 157 (32.2%) | 1582 (62.0%) | 112 (50.5%) |
| 13 to 24 | 5141 (34.0%) | 1105 (34.3%) | 109 (44.5%) | 184 (37.8%) | 783 (30.7%) | 67 (30.2%) |
| 24 or more | 2138 (14.1%) | 354 (11.0%) | 45 (18.4%) | 146 (29.9%) | 184 (7.2%) | 43 (19.4%) |
| History of Cardiovascular Disease | 2599 (17.2%) | 461 (14.3%) | 36 (14.7%) | 130 (26.7%) | 256 (10.0%) | 33 (14.9%) |
| Charlson Comorbidity Index |  |  |  |  |  |  |
| 1 | 12459 (96%) | 2822 (96%) | 209 (94%) | 301 (95%) | 2262 (98%) | 188 (96%) |
| 2 | 303 (2.3%) | 65 (2%) | 8 (4%) | 8 (3%) | 26 (1%) | 4 (2%) |
| 3+ | 203 (1.4%) | 44 (1%) | 6 (3%) | 8 (3%) | 14 (<1%) | 3 (1%) |
| HbA1c, mean (SD) | 8.8 (1.9) | 8.5 (1.5) | 8.8 (2.1) | 10.0 (2.9) | 8.6 (1.5) | 9.2 (2.4) |
| Systolic Blood Pressure, mean (SD) | 135.3 (15.4) | 134.3 (14.4) | 134.9 (15.3) | 132.1 (17.9) | 135.2 (14.6) | 133.3 (15.4) |
| eGFR, mean (SD) | 80.4 (26.5) | 88.0 (38.9) | 93.0 (23.9) | 83.5 (36.4) | 81.3 (24.0) | 80.2 (25.4) |
| Number of Unique Drugs |  |  |  |  |  |  |
| 0-5 | 12318 (81%) | 2716 (84%) | 197 (80%) | 392 (81%) | 2177 (85%) | 177 (80%) |
| 6-10 | 2799 (18.5%) | 504 (16%) | 48 (20%) | 95 (20%) | 372 (14%) | 45 (20%) |
| 11-15 | 8 (<1%) | 2 (<1%) | 0 (0%) | 0 (0%) | 0 (0%) | 0 (0%) |
| Statins | 11285 (75%) | 2536 (79%) | 170 (69%) | 295 (61%) | 2038 (80%) | 171 (77%) |
| Calcium Channel Blockers* | 4409 (30%) | 886 (28%) | 78 (32%) | 140 (29%) | 683 (27%) | 57 (26%) |
| Beta-Blockers | 3755 (25%) | 694 (21%) | 47 (19%) | 122 (25%) | 539 (21%) | 51 (23%) |
| Anticoagulants | 865 (6%) | 132 (4%) | 14 (6%) | 32 (6%) | 53 (2%) | 13 (5%) |
| Antiplatelets | 6131 (41%) | 1100 (34%) | 84 (34%) | 184 (37%) | 1077 (42%) | 88 (39%) |
| ACE/ARB/Renin | 8797 (58%) | 1873 (58%) | 154 (63%) | 229 (47%) | 1506 (59%) | 125 (56%) |
| Diuretics | 4705 (31%) | 853 (27%) | 87 (35%) | 153 (31%) | 651 (25%) | 78 (35%) |
